# Angiotensin II induces kidney inflammatory injury and fibrosis through binding to myeloid differentiation protein-2 (MD2)

**DOI:** 10.1038/srep44911

**Published:** 2017-03-21

**Authors:** Zheng Xu, Weixin Li, Jibo Han, Chunpeng Zou, Weijian Huang, Weihui Yu, Xiaoou Shan, Hazel Lum, Xiaokun Li, Guang Liang

**Affiliations:** 1 Chemical Biology Research Center, School of Pharmaceutical Sciences, Wenzhou Medical University, Wenzhou, Zhejiang, China; 2 The First Affiliated Hospital, Wenzhou Medical University, Wenzhou, Zhejiang, China; 3 The Second Affiliated Hospital and Yuying Children’s Hospital of Wenzhou Medical University, Wenzhou, Zhejiang, China

## Abstract

Growing evidence indicates that angiotensin II (Ang II), a potent biologically active product of RAS, is a key regulator of renal inflammation and fibrosis. In this study, we tested the hypothesis that Ang II induces renal inflammatory injury and fibrosis through interaction with myeloid differentiation protein-2 (MD2), the accessory protein of toll-like receptor 4 (TLR4) of the immune system. Results indicated that in MD2^−/−^ mice, the Ang II-induced renal fibrosis, inflammation and kidney dysfunction were significantly reduced compared to control Ang II-infused wild-type mice. Similarly, in the presence of small molecule MD2 specific inhibitor L6H21 or siRNA-MD2, the Ang II-induced increases of pro-fibrotic and pro-inflammatory molecules were prevented in tubular NRK-52E cells. MD2 blockade also inhibited activation of NF-κB and ERK. Moreover, MD2 blockade prevented the Ang II-stimulated formation of the MD2/TLR4/MyD88 signaling complex, as well as the increased surface binding of Ang II in NRK-52E cells. In addition, Ang II directly bound recombinant MD2 protein, rather than TLR4 protein. We conclude that MD2 is a significant contributor in the Ang II-induced kidney inflammatory injury in chronic renal diseases. Furthermore, MD2 inhibition could be a new and important therapeutic strategy for preventing progression of chronic renal diseases.

Chronic kidney diseases (CKD), a growing world-wide health problem, can progress to the serious end-stage kidney disease, in which patients require dialysis or a kidney transplant^1^,^2^. Much evidence now supports involvement of inflammation and the immune system as significant factors responsible for the renal tissue injury and remodeling found CKD. In patients with CKD, renal disease severity and inflammatory molecules directly correlate with toll-like receptors (TLRs) of innate immunity, particularly TLR4^3^,^4^. TLR4 is a versatile pattern recognition receptor of the innate immune system, and can be recognized and activated by pathogenic microbial products (e.g., LPS) or nonpathogenic endogenous agents (e.g., free fatty acids), producing a host of pro-inflammatory, anti-viral, and anti-bacterial cytokines^5^. At least for LPS signaling, LPS is required to bind MD2 (myeloid differentiation protein 2), which in turn forms a complex with TLR4, recruits adaptor proteins such as MyD88, resulting in activation of signaling cascades (i.e., NF-κB and ERK) for generation of a host of pro-inflammatory molecules^5^,^6^. A variety of experimental animal models support a crucial role of TLRs in the severity and progression of CKD. In mouse models of chronic kidney injury, the mutant non-functional TLR4^7^ or TLR4 knock-out^8^ protects against kidney dysfunction, inflammatory injury, and fibrosis. Cultures of proximal tubular epithelial cells with knock-down of TLR4 also presented with inhibition of NF-κB, a downstream signaling target of TLR4^9^,^10^. Despite the growing evidence for TLR4’s role as a key factor in mediating the inflammatory injury and fibrosis of CKD, the specific mechanism of activation of this potential pathogenic pathway of CKD remains unclear.

The inflammatory injury and tissue remodeling of CKD has also been attributed to angiotensin II (Ang II), the main peptide in the renin-angiotensin system (RAS) that plays a central role in the regulation of vascular tone, blood pressure, and electrolyte homeostasis. Ang II is known to be pro-inflammatory, directly activating renal glomerular and tubular cells as well as local immune cells to increase expression of cytokines (e.g., TGF-β, MCP-1), and inflammatory and fibrotic factors^1^. Classically, the actions of Ang II are believed to be mediated by its cognate Ang II type 1 (AT1) G protein-coupled receptor for regulation of both pressor and pro-inflammatory responses in kidney tissue^11^. However, growing evidence obtained from a variety of experimental models indicates that Ang II appears to also act through TLR4. Matsuda *et. al.*^12^ found that with TLR4-deficiency in a mouse model of cardiac dysfunction, Ang II-stimulated oxidative stress and MCP-1 expression are prevented. Similarly, in vascular smooth muscle cells, Ji and coworkers^13^ reported that in the presence of anti-TLR4 antibody, the Ang II-stimulated TNF-α secretion and MMP-9 are prevented. Recently, in a mouse model of CKD using Ang II infusion, mice with the mutant non-functional TLR4 are protected from developing albuminuria, elevated serum levels of BUN and creatinine, glomerulosclerosis, and interstitial fibrosis^7^. These findings suggest a mechanism by which Ang II signals downstream pro-inflammatory targets in a TLR4-dependent manner. Moreover, Ang II is hypothesized as a potential endogenous ligand of TLR4^14^, despite that it is unclear how Ang II interacts/activates TLR4.

MD2 is an extracellular glycoprotein essential for binding with LPS in the TLR4-induced inflammatory responses. Here, we tested the hypothesis that Ang II induces renal inflammatory injury and fibrosis through interaction with MD2, the accessory protein of TLR4 of the immune system. For study, the effects of Ang II-mediated renal inflammatory injury in CDK was investigated by MD2 blockade using genetic deletion of MD2 in mice (MD2^−/−^) or the small molecule specific inhibitor of MD2, L6H21^15^,^16^,^17^, in renal cells. Results indicated that MD2 blockade prevented Ang II-induced kidney dysfunction, upregulation of pro-inflammatory and pro-fibrotic molecules, and activation of NF-κB and ERK. Moreover, Ang II directly bound MD2, providing the potential mechanism of activation of the MD2/TLR4 signaling complex. We conclude that MD2 is a significant contributor in the Ang II-induced kidney tissue injury and remodeling, and could be a new and important therapeutic strategy for preventing progression of chronic renal diseases.

## Results

### MD2^−/−^ mice were protected from Ang II-induced renal dysfunction and tissue remodeling

**Figure 1:**
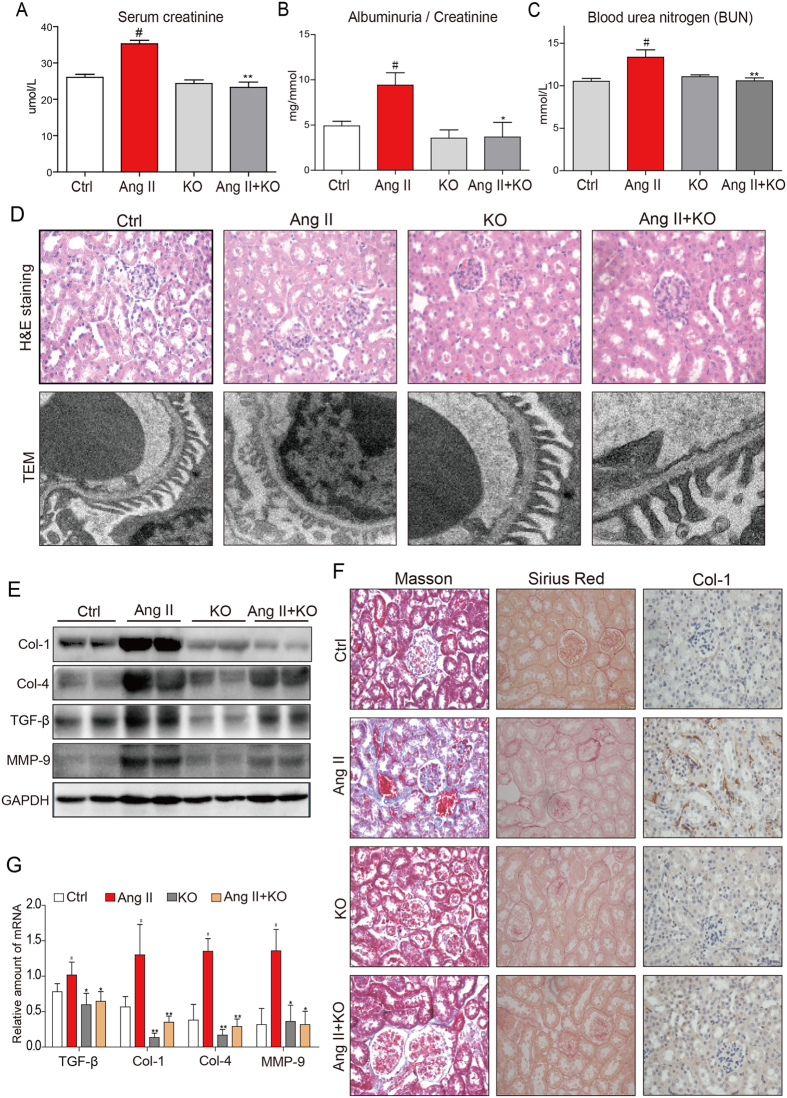
MD2^−/−^ mice were protected from Ang II-induced renal dysfunction and tissue remodeling. WT and MD2^−/−^ (KO) mice were injected subcutaneously with 1.4 mg·kg^−1^·day Ang II for 8 weeks, and blood and tissue samples were collected for analysis (Methods). Ctrl = vehicle injection in WT mice, Ang II = Ang II injecton in WT mice, KO = vehicle injection in MD2^−/−^ mice, Ang II + KO = Ang II injection in MD2^−/−^ mice; 8 mice/group. Kidney function indices, (**A**) serum creatinine level, (**B)** serum albumin/serum creatinine ratio and (**C)** blood urea nitrogen (BUN) in mmol/L. (**D**) Top row shows representative histological image kidney tissue from 5 mice per group (hematoxylin and eosin, 400x magnification); bottom row shows representative images from transmission electron microscopic (TEM) evaluation of renal tissue from each experimental group; 20000x magnification. (**E)** Representative Western blot analysis of kidney tissue for marker proteins of tissue modeling (Col = collagen, TGF-β = transforming growth factor β, and MMP-9 = matrix metalloproteinase 9; GAPDH as loading control; the densitometric quantification was shown in Supplementary Figure S1. The gels were run under the same experimental conditions. Shown are cropped gels/blots (The gels/blots with indicated cropping lines are shown in Supplementary Figure S6). (**F)** Representative histochemical images for renal tissue fibrosis from 5 mice per group evaluated by Masson’s trichrome staining (blue), Sirius red staining (red), and collagen 1 immunochemistry (Col-1)(brown); 400x magnification. (**G)** The mRNA expression of the TGF-β, Col1, Col4 and MMP-9 in renal tissue was determined by real-time qPCR; values were normalized to housekeeping gene β-actin. For data in (**A**,**B**,**G**), values are reported as mean ± S.E.M from 8 mice per group; ^#^P < 0.05 versus Ctrl; *P < 0.05, **P < 0.01 versus Ang II-treated group).

Our findings indicated that the subcutaneous injection of Ang II into mice resulted of renal dysfunction, as indicated by abnormal levels of serum creatinine, albuminuria (ALB)/creatinine ratio, and blood urea nitrogen (BUN) (Fig. 1A–C). However, in MD2^−/−^ mice, the Ang II-induced renal dysfunction was significantly reduced (Fig. 1A–C). We next evaluated the effects of Ang II on morphological changes of kidney structure in the control wild-type and MD2^−/−^ mice. Histological results indicated that Ang II induced primarily glomerular abnormalities, with disarrayed glomerular tufts, but these changes were not apparent in MD2^−/−^ mice (Fig. 1D). At the electron microscopic level, the Ang II-induced glomerular abnormalities were associated with unorganized shortened processes of podocytes, which were not apparent in the Ang II-treated MD2^−/−^ mice (Fig. 1D). We evaluated the extent of fibrosis induced by Ang II in the mouse kidney. Western blot analysis indicated that Ang II increased the content of Col-1, Col-4, TGF-β, and MMP-9, implicating kidney tissue remodeling (Fig. 1E and Supplementary Figure S1). This finding was corroborated by histochemical staining for collagen Type I (Col-1), Masson’s trichrome for collagen, and Sirius Red for fibrosis (Fig. 1F). In the MD2^−/−^ mice, the Ang II-induced fibrosis in the kidney was prevented (Fig. 1E and F). The protective effects against kidney fibrosis observed with MD2 knock-out was further evaluated by determining the mRNA of TGF-β, Col-1, Col-4, and MMP-9, important fibrotic tissue remodeling molecules. Results indicated that Ang II significantly increased expression of the four markers in mouse kidney tissue, but MD2 knockout prevented the increase (Fig. 1G). In mice injected with high doses of Ang II over a period of 8 weeks, the sustained hypertension should be induced and may contribute to the kidney injury. We then tested the effect of MD2 deficiency on blood pressure. As shown in the Supplementary Table S1, WT mice and MD2 knockout mice exhibited similar systolic blood pressure (SBP) before Ang II treatment. Subcutaneous Ang II infusion induced a significant increase in SBP in both WT and MD2^−/−^ mice, indicating that genetic knockout of MD2 could not affect the SBP level. Thus, the protection of MD2 knockout against kidney injury comes from other mechanism, i.e. anti-inflammation, rather than the change in loading condition.

### MD-2^−/−^ mice decreases Ang II-induced cytokines, ERK and NF-κB signaling

**Figure 2:**
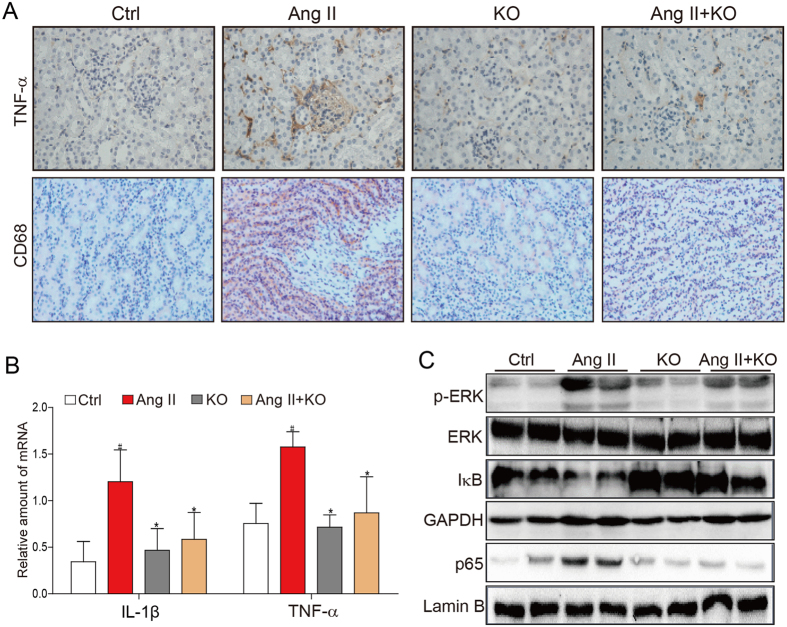
MD2^−/−^ mice have reduced inflammatory cytokine expression and signal activation in response to Ang II. WT and MD2^−/−^ (KO) mice were injected subcutaneously with 1.4 mg·kg^−1^·day Ang II for 8 weeks, and blood and tissue samples were collected for analysis (Methods). Ctrl = vehicle injection in WT mice, Ang II = Ang II injecton in WT mice, KO = vehicle injection in MD2^−/−^ mice, Ang II + KO = Ang II injection in MD2^−/−^ mice; 8 mice/group. (**A)** Representative immunohistochemical detection of TNF-α (brown) and CD68 (red) in renal tissue from 5 mice per group; 400x magnification. (**B)** The renal tissue mRNA levels of inflammatory cytokines, IL-1β and TNF-α were determined by real-time qPCR; values normalized to house-keeping gene β-actin and reported as mean ± S.E.M from 8 mice per group; *P < 0.05 versus Ang II-treated group, ^#^P < 0.05 versus Ctrl. (**C)** Representative Western blot analysis for p-ERK and IκB from renal tissue lysate (from 5 mice per group), and for NF-κB p65 subunit from nuclear lysates; GAPDH and lamin B served as loading controls, respectively. The gels were run under the same experimental conditions. Shown are cropped gels/blots (The gels/blots with indicated cropping lines are shown in Supplementary Figure S6).

Ang II is considered to be a pro-inflammatory factor, resulting in inflammation, macrophage infiltration, fibrosis, and accumulation of extracellular matrix. Therefore, we determined whether Ang II-induced inflammatory response observed in kidney tissue requires MD2. Immunohistochemical results indicated that Ang II increased the tissue content of TNF-α and CD68, a marker of infiltrated macrophages, but MD2 knockout prevented these increases (Fig. 2A). Moreover, Ang II significantly increased the mRNA level of IL-1β and TNF-α, and similarly, MD2 knockout prevented the increases (Fig. 2B). Both ERK and NF-κB are recognized downstream signaling targets of TLR4 which are critical regulators of inflammatory activity. Our results indicated that Ang II increased ERK phosphorylation and increased IκB degradation of kidney tissue, indication activation of these signaling molecules (Fig. 2C). Moreover, Western blot analysis of the p65 subunit of NF-κB from the nuclear cell fraction indicated that Ang II increased p65 nuclear translocation (Fig. 2C). However, kidney tissue from Ang II-treated MD2^−/−^ mice showed reduced ERK phosphorylation, IκB degradation, and p65 nuclear translocation (Fig. 2C). These results indicated that the pro-inflammatory activity of Ang II were MD2-dependent.

### MD2-dependency of Ang II-induced pro-fibrotic and pro-inflammatory responses in renal tubular epithelial cells

**Figure 3:**
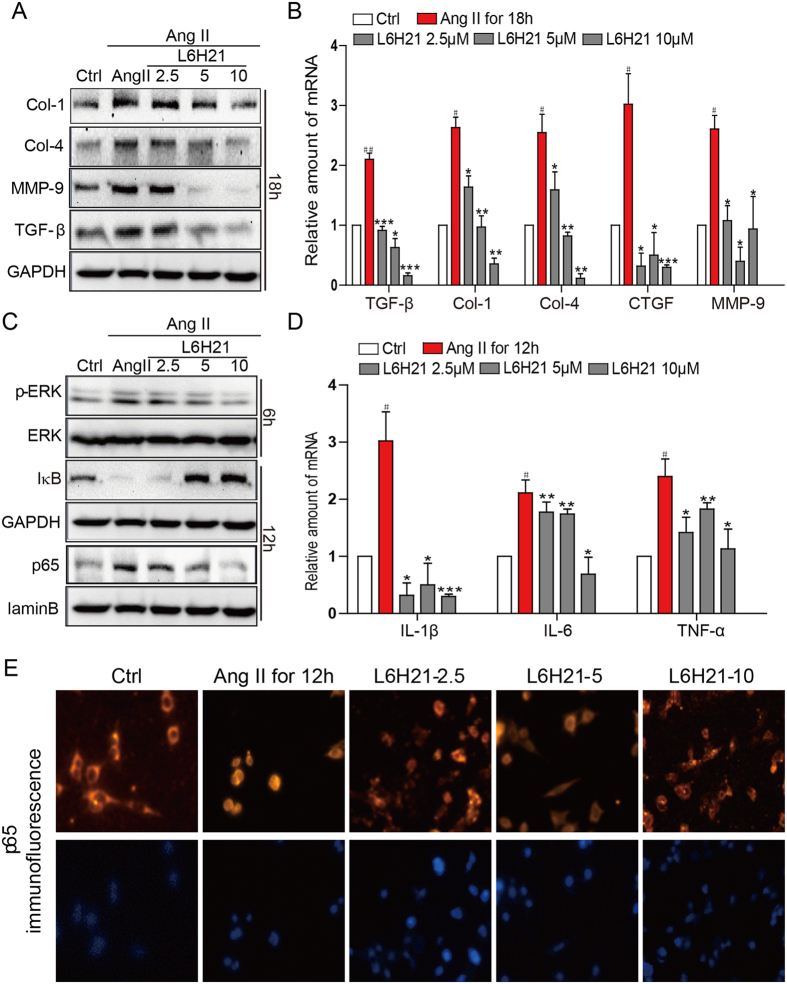
L6H21 pretreatment prevents Ang II-induced fibrosis and signal activation in renal tubular epithelial cells. The effects of the MD2 inhibitor L6H21 on Ang II-stimulated inflammatory responses in the renal tubular epithelial cell line, NRK-52E, were determined. NRK-52E were pretreated with the vehicle control DMSO (Ctrl) or L6H21 (2.5, 5.0, 10 μM) for 1 h, and stimulated with Ang II (1 μM) for different periods. (**A)** Representative Western blot analysis for protein markers of fibrosis, Col-1 (collagen 1), Col-4 (collagen 4), MMP-9 (metalloproteinase 9), and TGF-β (transforming growth factor β), GAPDH as loading control; n = 3 independent determinations. (**B)** The mRNA levels of TGF-β, Col-1 (collagen 1), Col-4, CTGF, and MMP-9 were detected by real-time qPCR; values normalized to house-keeping gene β-actin. (**C)** Representative Western blot analysis for p-ERK (phosphorylated ERK) and IκB from total cell lysate, GAPDH as loading addingol, ERK = total ERK; NF-κB p65 subunit detected from nuclear cell fraction, lamin B as loading control; n = 3 independent determinations. (**D)** The mRNA levels of IL-1β, IL-6 and TNF-α were detected by real-time qPCR; values normalized to house-keeping gene β-actin. (**E)** Representative immunofluorescent distribution of NF-κB p65 subunit (Texas-red streptavidin), top row; same cells counterstained with nuclear stain DAPI, bottom row, 200x magnification; n = 3 independent determinations. Data in B and D are reported as mean ± S.E.M. of n = 3, *P < 0.05, **P < 0.01, ***P < 0.001 versus Ang II-treated group; ^#^P < 0.05 and ^##^P < 0.01 versus Ctrl. For panels (**A**,**C**) the gels were run under the same experimental conditions. Shown are cropped gels/blots (The gels/blots with indicated cropping lines are shown in Supplementary Figure S6).

We next investigated the MD2-dependency of inflammatory responses at the cellular level. Rat tubular epithelial cells, NRK-52E, were direct stimulated with Ang II (1 μM) with or without pretreatment with the specific MD2 inhibitor, L6H21, and determined expression of pro-fibrotic molecules. Results indicated that Ang II increased production of Col-1, Col-4, TGF-β and MMP-9 proteins, but pretreatment with L6H21 dose-dependently inhibited the increased expression of these proteins (Fig. 3A). At the mRNA level, Ang II significantly increased TGF-β, Col-1, Col-4, CTGF (connective tissue growth factor) and MMP-9, and L6H21 pretreatment effectively prevented these increases in mRNA expression (Fig. 3B).

The downstream targets of MD2/TLR4, ERK and NF-κB, are known to signal regulation of a host of pro-fibrotic and pro-inflammatory molecules. Western blot results indicated that Ang II increased ERK phosphorylation and degradation of IκB-α, as well as increased nuclear distribution of NF-κB p65 subunit, and the pretreatment with L6H21 effectively prevented these Ang II-mediated effects (Fig. 3C). With immunofluorescence detection, Ang II was observed to increase the nuclear translocation of NF-κB p65 subunit from the cytoplasm (Fig. 3E), supporting activation of NF-κB. However, when the cells were pretreated with L6H21, the Ang II-induced translocation of NF-κB p65 subunit was inhibited (Fig. 3E). We next determined the inflammatory responses in NRK-52E cells challenged with Ang II by measuring key pro-inflammatory cytokines. Results indicated that Ang II induced 2–3-fold increases of IL-1β, IL-6, and TNF-α mRNA (Fig. 3D). With L6H21 pretreatment, the Ang II-induced increase in IL-1β mRNA was dramatically prevented, with expression below control levels (Fig. 3D). The L6H21 pretreatment was also effective in inhibition of the Ang II-induced increases of IL-6 and TNF-α mRNA (Fig. 3D). These data indicated that the increased pro-fibrotic and pro-inflammatory activities in response to Ang II challenge required MD2.

### Ang II signals through the MD2/TLR4 pathway

**Figure 4:**
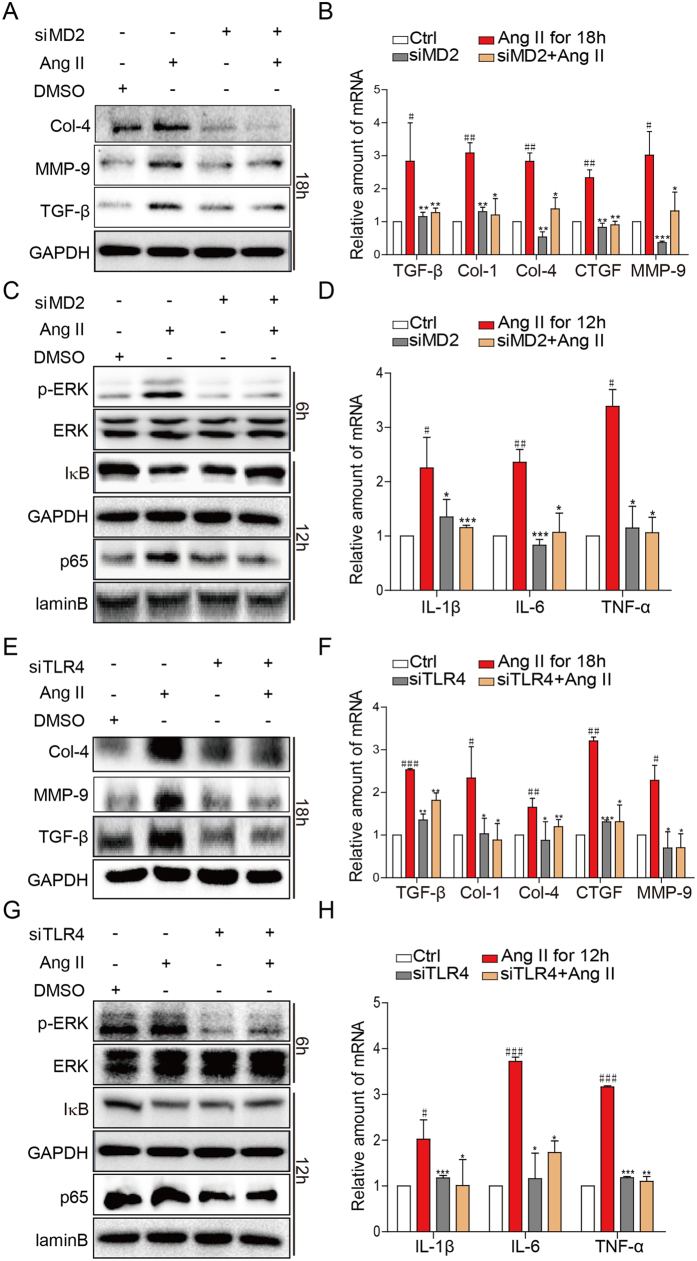
Ang II-induced inflammatory responses and signaling activation are dependent on the MD2 and TLR4. NRK-52E cells were transfected with 1 μg MD2- or TLR4-specific siRNA in medium for 24 h, and stimulated with Ang II (1 mM) for different periods. (**A,E)** Representative Western blot analysis for marker proteins of fibrosis: Col-4 (collagen 4), MMP-9 (metalloproteinase 9), TGF-β (transforming growth factor β), GAPDH as loading control, n = 4 independent determinations. (**B,F)** The mRNA levels of TGF-β, Col-1 (collagen 1), Col-4, CTGF, MMP-9 were measured by real-time qPCR; values normalized to house-keeping gene β-actin. (**C,G)** Representative Western blot analysis for phosphorylated ERK (p-ERK), total ERK, and IκB from cell lysate; NF-κB p65 subunit analyzed from nuclear cell fraction; GAPDH and lamin B served as respective loading controls; n = 4 independent determinations. **D,H)** The mRNA levels of IL-1β, IL-6 and TNF-α were detected by real-time qPCR, values normalized to house-keeping gene β-actin. For data in (**B**,**D**,**F**, and **H**) values are reported as mean ± S.E.M. of n = 3; *P < 0.05, **P < 0.01, ***P < 0.001 versus Ang II-treated group; ^#^P < 0.05, ^##^P < 0.01, ^###^P < 0.001 versus Ctrl. For panels A, C, E, and G, the gels were run under the same experimental conditions. Shown are cropped gels/blots (The gels/blots with indicated cropping lines are shown in Supplementary Figure S6).

Our finding of MD2 dependency in the Ang II-induced responses implicated involvement of the MD2/TLR4 complex. We investigated the role of the MD2/TLR4 complex in the Ang II-induced pro-fibrotic and pro-inflammatory activities by using siRNA-mediated knockdown of MD2 or TLR4. NRK-52E cells were transfected with siRNA targeted to MD2 (siMD2), TLR4 (siTLR4), or negative control sequence (see Methods), challenged with Ang II, and cells collected for determination of pro-fibrotic, pro-inflammatory, and signaling molecules as described before. The siRNA silencing remarkably decreased MD2 and TLR4 expression, respectively (Supplementary Figure S2) Results indicated that in siMD2 cells, the Ang II-induced increases of pro-fibrotic molecules (protein and mRNA) (Fig. 4A and B, respectively), activation of ERK and NF-κB (Fig. 4C), and increases of pro-inflammatory cytokines IL-1β, IL-6, TNF-α (Fig. 4D) were all significantly inhibited. Predictably, in siTLR4 cells, the Ang II-induced increases of pro-fibrotic molecules (protein and mRNA) (Fig. 4E and F, respectively), activation of ERK and NF-κB (Fig. 4G), and increases of pro-inflammatory cytokines IL-1β, IL-6, TNF-α (Fig. 4H) were also significantly inhibited. The findings provide support that the Ang II-mediated responses required both MD2 and TLR4, implicating dependency for the MD2/TLR4 complex.

### Ang II induces MD2/TLR4 complex formation and interacts MD2

**Figure 5:**
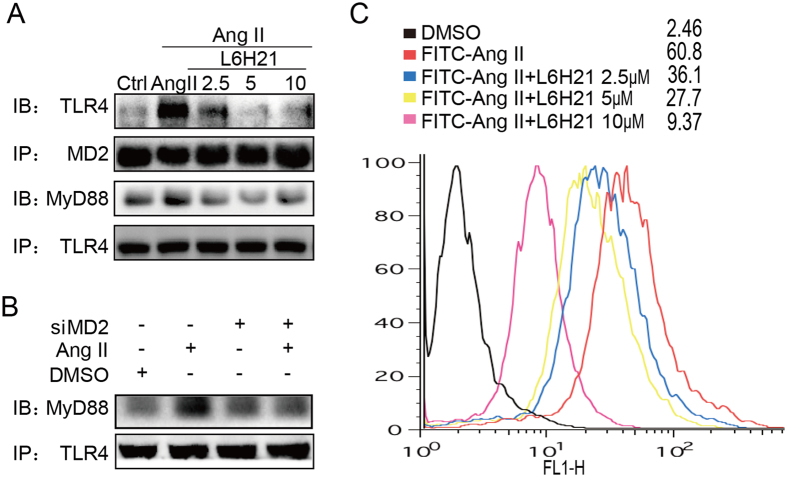
Ang II induces MD2/TLR4 complex formation in renal cells. Effects of MD2 blockade by (**A)** L6H21 or (**B)** siRNA-mediated knock-down on Ang II-induced MD2/TLR4 complex formation were determined. NRK-52E cells were pretreated with L6H21 (2.5, 5, 10 μM) for 1 h, or transfected with 1 μg MD2-specific siRNA (Methods), stimulated with Ang II (1 mM) for 30 min, and cell lysates were co-immunoprecipitated (IP) with anti-MD2 or -TLR4 antibodies, and Western blot analysis (IB) made to detect TLR4 or MyD88; n = 4 independent determinations. The gels were run under the same experimental conditions. Shown are cropped gels/blots (The gels/blots with indicated cropping lines are shown in Supplementary Figure S6). (**C**) The effects of L6H21 on Ang II binding to cell surface was determined. NRK-52E cells were incubated with Ang II conjugated with fluorescent FITC (FITC-Ang II; 50 mg/ml) with or without L6H21 (2.5, 5.0, 10 μM), and flow cytometry was used to detect cell-bound fluorescence; shown is a representative flow analysis with the median fluorescence intensity (MFI) indicated for each group; n = 4 independent determinations.

The specific mechanisms by which Ang II activated the MD2/TLR4 signaling complex are not known, but would be important to understand for future therapeutic target design. We first determined the effects of the MD2 inhibitor, L6H21, on the Ang II-induced MD2/TLR4 complex formation in NRK-52E cells. Co-immunoprecipitation results indicated that with increasing L6H21 concentration of 2.5–10.0 μM, decreasing amounts of TLR4 was detected in the immunocomplex, with a corresponding decreased recruitment of the key adaptor protein, MyD88 (Fig. 5A). A subsequent co-immunoprecipitation study using siMD2 cells indicated similar results, in that the Ang II-induced recruitment of MyD88 was impaired (Fig. 5B). The findings suggested that a functional MD2 was required for Ang II to activate the MD2/TLR4 complex.

Data in Supplementary Table S1 has excluded the effect of hypertension on MD2-dependent renal inflammation *in vivo*. Here, we further confirmed this note using an Ang II receptor blocker (irbesartan, IRB). The Supplementary Figure S3A–C showed that, compared with L6H21, IRB failed to block Ang II-induced MD2/TLR4 complex formation and inflammatory cytokine expression.

In addition to tubular cells, other renal cell types (i.e. glomerular mesangial cells), infiltrated macrophages, and endothelial cells located within the glomeruli or in peritubular capillaries are also immunologically active and may also play a key role in the progression of Ang II-induced CKD. We then determined MD2/TLR4 complex and inflammation in Ang II-stimulated renal glomerular mesangial SV40 cells, human microvascular endothelial cells (HMEC-1), and mouse primary macrophages (MPMs, as infiltrating leukocytes). These data were shown in the Supplementary Figure S4A–H. It was observed that MD2/TLR4 are also expressed and Ang II induced the formation of MD2/TLR4 complex and TLR4/MyD88 complex in SV40 and HMEC-1 cells. Macrophage is a well-known cell type that expresses MD2 and TLR4 to respond endotoxin LPS innate immunity. In all three cell types, Ang II induced inflammatory cytokine release, which is inhibited by MD2 inhibitor L6H21 (Supplementary Figure S4B,C and E–H).

We further investigated the effects of L6H21 on *in situ* Ang II binding on the cell surface of NRK-52E cells. The incubation of NRK-52E cells with FITC-labelled Ang II (FITC-Ang II) resulted in increased surface fluorescence detected by flow cytometry with an MFI (mean fluorescence index) of 60.8 (Fig. 5C). However, treatment with L6H21 at increasing concentrations progressively inhibited the MFI (Fig. 5C), indicating that L6H21 interfered with FITC-Ang II binding onto the cell surface. We have previously reported that Ang II is able to bind MD2 directly^18^. Ang II may initially interact with MD2 directly, in a similar manner as with LPS, in order to activate MD2/TLR4 complex formation. Here, we further confirmed the previous conclusion using the methods as described in the supplementary file. The ELISA system showed that biotin-labelled Ang II (biotin-Ang II) bound to recombinant human MD2 (rhMD2) in a dose-dependent manner (Supplementary Figure S5a), while L6H21 was able to dose-dependently inhibited the biotin-Ang II binding (100 μM) with rhMD2 (Supplementary Figure S5B). Surface plasmon resonance (SPR) spectroscopy further evaluated Ang II binding with MD2 and TLR4. In consistent with previous result^18^, Ang II bound rhMD2 with a high affinity (Supplementary Figure S5C), but failed to bind recombinant human TLR4 protein under the same experimental condition (Supplementary Figure S5D). These data further validate that MD2, rather than TLR4, is the direct target of Ang II in the kidney. Ang II binds onto MD2 as an initial step to drive the subsequent formation and activation of the MD2/TLR4 complex.

## Discussion

CKD is a recognized world-health concern, but the underlying basis for its severity and progression to the more serious end-stage renal disease remains poorly understood. In this study, we tested the hypothesis that Ang II, the main RAS peptide, induces renal inflammatory injury and fibrosis through interaction with MD2, the accessory protein of TLR4 of the immune system. Results indicated that Ang II-induced inflammatory injury, fibrosis, and signaling were MD2-dependent in mouse kidney tissue and kidney tubular epithelial cells. Findings provide strong evidence that the MD2-TLR4 signaling complex plays a predominant role in the Ang II-induced inflammatory injury and fibrosis in the CKD.

Our mouse model using 8 weeks of Ang II infusion resulted in significant elevations of serum creatinine, albuminuria/creatinine ratio, and serum BUN level, indicative of kidney dysfunction. The kidney dysfunction was accompanied by light and electron microscopic morphological evidence of glomerular damage, as well as increases of several indicators of tissue remodeling and fibrosis. These pathological parameters resemble those occurring in human CKD. Significantly, the Ang II-induced kidney dysfunction, renal tissue morphological damage, and increases of biochemical markers of fibrosis/tissue remodeling were not observed in the MD2^−/−^ mice. The findings are consistent with the report by Souza and coworkers^7^, who also used Ang II infusion of a mouse model, but expressing nonfunctional mutant TLR4, and concluded that TLR4 mutant mice were significantly protected from CKD progression. The similar findings obtained from the MD2^−/−^ or mutant TLR4 mice support MD2/TLR4 as a critical signaling complex in regulation of the progression and severity of CKD. Recent paper demonstrated that the progression toward CKD related to tissue fibrosis is associated with cell cycle arrest of kidney resident cells with the consequent production of profibrotic mediators^19^. Here, we failed to determine the cell cycle biomarkers in inflammation-mediated renal fibrosis, which may be a limitation of this paper.

We found that in kidney tubular epithelial cells, Ang II induced upregulated expression of pro-inflammatory genes, pro-fibrotic genes, and activated signaling targets ERK and NF-κB, all of which were inhibited by the small molecule MD2 inhibitor L6H21. The MD2-dependency of these findings suggests that the MD2/TLR4 complex was mobilized in signaling the inflammatory responses in kidney tubular epithelial cells, mesangial cells, macrophages, and endothelial cells. In support of this conclusion, the siRNA-mediated knock-down of TLR4^9^ or the presence of a TLR4 inhibitor^10^, prevented the TLR4 ligand-induced NF-κB activation in kidney tubular epithelial cells. Thus, the overall MD2-dependent inflammatory injury and fibrosis in CKD was attributed, in part, to the tubular epithelial cell population in the kidney. Nonetheless, not only tubular epithelial cells express TLR4, but also mesangial cells, podocytes, Bowman’s capsule, and infiltrating leukocytes^14^. Interestingly, we also found that MD2 and TLR4 are expressed in glomerular mesangial cells, macrophages, and endothelial cells. Ang II can induce MD2/TLR4/MyD88 signaling pathway activation and cytokine expression in these cell types, indicating that, in general, renal cells are immunologically active. Thus, likely different kidney cell populations contribute to the progression of CKD severity through MD2/TLR4 signaling.

Our identification of MD2 in the regulation of the Ang II-induced inflammatory injury provides a potential new mechanism in activating TLR4 in the pathogenesis of CKD. The present data and previously reported evidence^18^ from cell-free assays supported direct binding of Ang II with MD2. This direct interaction of Ang II with MD2 was analogous to LPS binding with MD2, resulting in MD2/TLR4 complex formation, and recruitment of adaptor molecules for signaling. In future, the structural biology studies on Ang II/MD2 complex co-crystal are urgently needed to demonstrate the precise binding mode. Consistent with this mechanism of TLR4 activation, our results from kidney tubular epithelial cells indicated that Ang II stimulated MD2/TLR4 complex formation and recruitment of MyD88, a major adaptor protein of TLRs, including TLR4, that is essential for signaling to ERK and NF-κB^5^. MD2 was crucial for activation of this signaling cascade since the presence of L6H21 or knock-down of MD2 prevented recruitment of MyD88, activation of ERK and NF-κB, and consequent upregulation of pro-inflammatory and pro-fibrotic molecules in response to Ang II. Not surprisingly, similar results were obtained with knock-down of TLR4. Thus, Ang II-induced renal tissue inflammatory injury was regulated through a mechanism of direct binding of Ang II with MD2, resulting in MD2/TLR4 complex formation and activation. In addition, the classic Ang II receptors are the best characterized to mediate the effects of Ang II, including vasopressor effects and aldosterone secretion. Few studies have addressed the role of local AT1 receptor in the pro-inflammatory activities of Ang II. Here, we found that blocking AT1 receptor using IRB was not able to attenuate Ang II-induced MD2/TLR4 complex formation and TNF-α/IL-6 production. And, MD2 deficiency did not affect the hypertension in mice. These findings indicate that AT1-mediated hypertension and MD2-mediated inflammation seem to be independent on each other. Future studies would be valuable in precisely elucidating the different signaling pathways of Ang II for the treatment of related diseases.

The small molecule MD2 inhibitor, L6H21, appears to inhibit MD2 through competition with Ang II binding on MD2. We observed that increasing L6H21 concentrations were able to decrease the biotin-conjugated Ang II bound on rhMD2. Similarly, the amount of FITC-conjugated Ang II bound to the cell surface was decreased with increasing L6H21 concentrations. The findings suggest that L6H21 binding to MD2 displaced Ang II binding on MD2, although it remains to be determined whether L6H21 occupy similar sites as Ang II within the MD2 pocket. The mechanism of inhibition by L6H21 will be of great interest since the inhibitor has shown to be highly effective as an anti-inflammatory agent in experimental models of sepsis^16^ and diabetic nephropathy^17^.

In summary, results indicated that Ang II induced MD2-dependent kidney dysfunction, morphological injury, upregulation of pro-inflammatory and pro-fibrotic molecules, and activation of NF-κB and ERK. Moreover, the mechanism of regulation engaged direct Ang II binding with MD2, triggering subsequent signaling cascades responsible for the inflammatory responses. We conclude that MD2 is a significant contributor in the Ang II-induced kidney tissue injury and remodeling in chronic renal diseases. Furthermore, MD2 inhibition could be a new and important therapeutic strategy for preventing progression of chronic renal diseases.

## Materials and Methods

### Cell culture

The rat tubular epithelial cell line NRK-52E was obtained from the Shanghai Institute of Biochemistry and Cell Biology (Shanghai, People’s Republic of China). The cells were cultured in Dulbecco’s modified Eagle’s medium/Ham’s F12 medium and 25 mM (high-glucose) D-glucose supplemented with 5% fetal bovine serum, 100 U/ml penicillin, and 100 U/ml streptomycin and maintained at 37 °C in humidified 5% CO_2_. For study, cells were pretreated for 1 h with L6H21 at 2.5, 5.0, and 10.0 μM. L6H21 was dissolved in DMSO, and vehicle control cells received equal concentration of DMSO (dimethyl sulfoxide). The cells were then incubated with Ang II (1 mM) for 6 h, and used for the respective studies described.

### Materials

Ang II and irbesartan were purchased from Sigma- Aldrich (St. Louis, MO). Ang II labelled with FITC was purchased from Bankpeptide Biological Technology Co. (Hefei, Anhui province, China). Antibodies directed against p-ERK (phosphorylated ERK), ERK, NF-κB p65 subunit, laminB and I-κB were purchased from Cell Signaling Technology (Beverly, MA); anti-MD2 antibody was purchased from eBioscience (San Diego, CA, USA). The antibodies directed against TGF-β (transforming growth factor β), MMP-9 (metalloproteinase 9), collagen IV (Col-4), and GAPDH (glyceraldehyde 3-phosphate dehydrogenase) were purchased from Santa Cruz Biotechnology (Dallas, TX). L6H21 was synthesized and structurally identified using MS and 1H NMR analyses, as described by us^15^. For use in the biological experiments, L6H21 was recrystallized from CHCl_3_/EtOH, with an HPLC purity of >99%. In *in vitro* experiments, L6H21 was dissolved in DMSO, and DMSO alone served as vehicle control.

### Animals

The 6-week-old male C57BL/6 (WT) and MD2^−/−^ mice (n = 32) weighing 18–22 g were obtained from the Animal Centre of Wenzhou Medical University (Wenzhou, People’s Republic of China). The mice were housed with a 12-h light/dark cycle at a constant room temperature, fed with a standard rodent diet, and provided with free access to water. The mice were acclimated to the laboratory for at least 2 weeks before use for study. All animal care and experimental procedures complied with the “Detailed Rules and Regulations of Medical Animal Experiments Administration and Implementation” (Order No. 1998–55, Ministry of Public Health, People’s Republic of China) were approved by the Wenzhou Medical University Animal Policy and Welfare Committee. Protocols involving the use of animals were approved by the Wenzhou Medical University Animal Policy and Welfare Committee. Renal fibrosis was induced in mice by daily subcutaneous injections of Ang II (1.4 mg·kg^−1^·day in phosphate buffer, pH 7.2) for 8 weeks. The mice were randomly divided into 4 groups with 8 mice in each group: 1) control group (WT + PBS); 2) WT + Ang II; 3) MD2^−/−^ + PBS; 4) MD2^−/−^ + Ang II. The systolic blood pressure (SBP) in mice was detected at day 0, week 4^th^ and week 8^th^ after Ang II treatment by non-invasive tail-cuff Pressure Analysis System (Softron BP-98A, Tokyo, Japan). After 8 weeks of treatment, the mice were sacrificed under ether anesthesia, and the blood and kidney samples were collected analyses described below.

### Quantitative real-time qPCR

Total RNA was isolated from cells or tissues (50–100 mg) using TRIZOL (Invitrogen, Carlsbad, CA, USA). Reverse transcription and quantitative PCR (RT-qPCR) were performed using M-MLV Platinum RT-qPCR Kit (Invitrogen). Real-time quantitative PCR was carried out using the Eppendorf Realplex4 instrument (Eppendorf, Hamburg, Germany). Primers of genes, i.e., TNF-α, IL-6, IL-1β, Col-1, Col-4, CTGF, MMP-9, TGF-β and β-actin were synthesized from Invitrogen (Invitrogen, Shanghai, China). The primer sequences used are shown in the Supplementary Table S2. The relative amount of each gene was normalized to the amount of β-actin.

### Western blotting

Cells or renal tissue samples (30–50 mg) were lysed, and the protein concentrations were determined by using the Bradford protein assay kit (Bio-Rad Laboratories, Hercules, CA). Aliquots (about 100 mg of cellular protein) were subjected to electrophoresis and transfer to polyvinylidene fluoride membranes, which were then blocked in Tris-buffered saline containing 0.05% Tween 20 and 5% nonfat milk. The polyvinylidene fluoride membrane was incubated overnight with primary specific antibodies, followed by incubation with the appropriate secondary antibodies. The immunoreactive proteins were visualized with chemiluminescence (Bio-Rad Laboratories) reagent and quantitated by densitometry using Image J analysis software (version 1.38e; http://imagej.nih.gov/ij/), and were normalized to their respective control.

### Histologic analyses

Kidney tissue samples were fixed in 4% paraformaldehyde and embedded in paraffin. The paraffin sections (5 μm) were stained with hematoxylin and eosin or evaluation of general morphology, or with Masson’s trichrome (Nanjing KerGEN Bioengineering Institute, Jiangsu, People’s Republic of China) or Sirius Red for evaluation of fibrosis. The sections were viewed under a light microscope (400x magnification; Nikon, Tokyo, Japan).

### Electron microscopy

After sacrificed, the kidneys were collected and fixed in phosphate buffer (pH 7.4) containing 2.5% glutaraldehyde overnight at 4 °C. The tissues were postfixed in 1% OsO4 at room temperature for 60 min, stained with 1% uranyl acetate, dehydrated through graded acetone solutions, and embedded in Epon. Aeras containing tissues were block mounted and cut into 70-nm sections, and examined with the electron microscope (H-7500, Hitachi, Ibaraki, Japan).

### Immunohistochemistry

The renal sections (5 μm) were deparaffinized and rehydrated, and then subjected to antigen retrieval in 0.01 M citrate buffer (pH 6.0) by microwaving. After blocking with 5% bovine serum albumin, the sections were incubated with anti-collagen IV antibody (1:500) or anti-TNF-α antibody (1:1000) overnight at 4 °C, followed by the secondary antibody (1:100). The nucleus was stained with 4′,6-diamidino-2-phenylindole, and sections were viewed under a fluorescence microscope (400x magnification; Nikon).

### Measurements of serum biomarkers

The components of serum including the albumin, creatinine, and urine nitrogen, were detected using commercial kits (Nanjing Jiancheng Bioengineering Institute, Jiangsu, People’s Republic of China).

### Short interfering RNA transfection

The short interfering RNA (siRNA) duplexes and the Negative Universal Control sequences used in this study were purchased from Invitrogen (Carlsbad, CA, USA) and have the following sequences: MD2 target CCAUAUUUACUGAAU-CUGA beginning at nt 36, 5′-CCAUAUUUACUGAAUCUGATT-3_ (sense), 5′-UCAGAUUCAGUAAAUAUGGGA-3′ (antisense) (NCBI accession no. NM_001024279.1). TLR4 target CCGAUGCAAUUAUU UCCUA beginning at nt 81, 5_-CCGAUGCAAUUAUUUCCUATT-3 (sense), 5_-UAGGAAAUAAUUGCAUCGGAG-3_ (antisense) (NCBI accession no.NM_019178.1). Negative Universal Control sequence was used as the control. siRNA (1 μg) was mixed with 3 μl Lipofectamine RNAi-MAX in 200 μl of serum-free Opti-MEM and incubated for 20 min at room temperature. The mix was incubated with NRK-52E in Dulbecco’s modified Eagle’s medium (fetal bovine serum without antibiotics) and incubated for 24 h.

### Assay of cellular NF-κB p-65 translocation

The cells were immunofluorescence-labelled according to the manufacturer’s instruction using a Cellular NF-κB p65 Translocation Kit (Beyotime Biotech, Nantong, China). P65 protein and nuclei fluorescence as red and blue, respectively, and can be simultaneously viewed by fluorescence microscope (200x, Nikon, Tokyo, Japan) at an excitation wavelength of 350 nm for DAPI (4′,6-diamidino-2-phenylindole) and 540 nm for cyanine 3 (Cy3).

### Immunoprecipitation

TLR4 was co-precipitated with MD2 or MyD88 from NRK-52E cells to evaluate the effects of Ang II in the presence or absence of MD2 blockade on complex formation. NRK-52E cells were treated with Ang II 30 min after L6H21 pretreatment or siRNA to MD2. Cell extracts were incubated with anti-MD2 or anti-TLR4 antibody for 1 h and precipitated with protein G-Sepharose beads at 4 °C overnight. Western blot analysis was made from the supernatant to detect TLR4, MD2, and MyD88.

### Flow cytometric analysis

Cell surface binding of fluorescein isothiocyanate-labelled Ang II was measured as described in previous studies^20^. Briefly, NRK-52E cells (1 × 10^5^/ml) were incubated with Ang II-FITC (50 μg·mL^−1^) for 30 min with or without L6H21. After being washed, the cells bound with Ang II-FITC were quantified by flow cytometry.

### Statistical analysis

Data are presented as the mean ± S.E.M. The statistical significance between groups was obtained by Student’s t-test or ANOVA multiple comparisons in GraphPad Pro 5.0 (GraphPad, San Diego, CA, USA). Differences between experimental groups were considered to be significant at p < 0.05.

## Additional Information

**How to cite this article**: Xu, Z. *et al*. Angiotensin II induces kidney inflammatory injury and fibrosis through binding to myeloid differentiation protein-2 (MD2). *Sci. Rep.* **7**, 44911; doi: 10.1038/srep44911 (2017).

**Publisher's note:** Springer Nature remains neutral with regard to jurisdictional claims in published maps and institutional affiliations.

## Supplementary Material

Supplementary Information
