# Author Correction: Angiotensin II induces kidney inflammatory injury and fibrosis through binding to myeloid differentiation protein-2 (MD2)

**DOI:** 10.1038/s41598-021-94987-8

**Published:** 2021-07-29

**Authors:** Zheng Xu, Weixin Li, Jibo Han, Chunpeng Zou, Weijian Huang, Weihui Yu, Xiaoou Shan, Hazel Lum, Xiaokun Li, Guang Liang

**Affiliations:** 1 Chemical Biology Research Center, School of Pharmaceutical Sciences, Wenzhou Medical University, Wenzhou, Zhejiang China; 2 The First Affiliated Hospital, Wenzhou Medical University, Wenzhou, Zhejiang China; 3 The Second Affiliated Hospital and Yuying Children’s Hospital of Wenzhou Medical University, Wenzhou, Zhejiang China

Correction to: *Scientific Reports* https://doi.org/10.1038/srep44911, published online 21 March 2017

This Article contains errors in Figures 1 and 3.

In Figure 1D, the TEM images of the KO group and the AngII + KO group are incorrect. In Figure 3A, the GAPDH blot is a duplication of the GAPDH blot in Figure 3C. Additionally, in Figure 3C, the IκB and the Lamin B blots are incorrect.

The correct Figures 1 and 3 and accompanying legends appear below.

**Figure 1:**
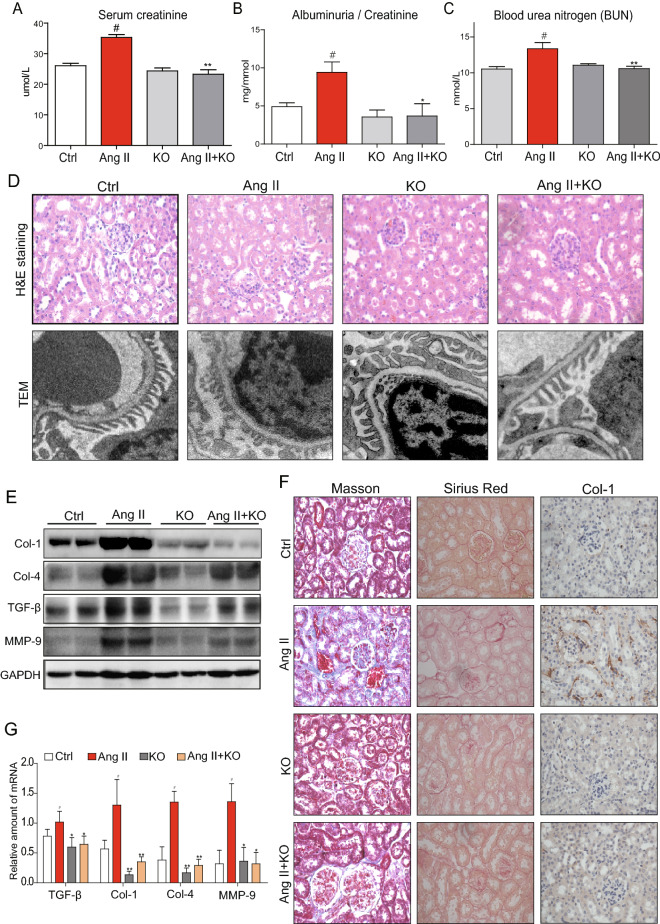
MD2^−/−^ mice were protected from Ang II-induced renal dysfunction and tissue remodeling. WT and MD2^−/−^ (KO) mice were injected subcutaneously with 1.4 mg·kg^−1^·day Ang II for 8 weeks, and blood and tissue samples were collected for analysis (Methods). Ctrl = vehicle injection in WT mice, Ang II = Ang II injecton in WT mice, KO = vehicle injection in MD2^−/−^ mice, Ang II + KO = Ang II injection in MD2^−/−^ mice; 8 mice/group. Kidney function indices, (**A**) serum creatinine level, (**B**) serum albumin/serum creatinine ratio and (**C**) blood urea nitrogen (BUN) in mmol/L. (**D**) Top row shows representative histological image kidney tissue from 5 mice per group (hematoxylin and eosin, 400× magnification); bottom row shows representative images from transmission electron microscopic (TEM) evaluation of renal tissue from each experimental group; 20,000× magnification. (**E**) Representative Western blot analysis of kidney tissue for marker proteins of tissue modeling (Col = collagen, TGF-β = transforming growth factor β, and MMP-9 = matrix metalloproteinase 9; GAPDH as loading control; the densitometric quantification was shown in Supplementary Figure S1. The gels were run under the same experimental conditions. Shown are cropped gels/blots (The gels/blots with indicated cropping lines are shown in Supplementary Figure S6). (**F**) Representative histochemical images for renal tissue fibrosis from 5 mice per group evaluated by Masson’s trichrome staining (blue), Sirius red staining (red), and collagen 1 immunochemistry (Col-1)(brown); 400× magnification. (**G**) The mRNA expression of the TGF-β, Col1, Col4 and MMP-9 in renal tissue was determined by real-time qPCR; values were normalized to housekeeping gene β-actin. For data in (**A**,**B**,**G**), values are reported as mean ± S.E.M from 8 mice per group; ^#^P < 0.05 versus Ctrl; *P < 0.05, **P < 0.01 versus Ang II-treated group).

**Figure 3:**
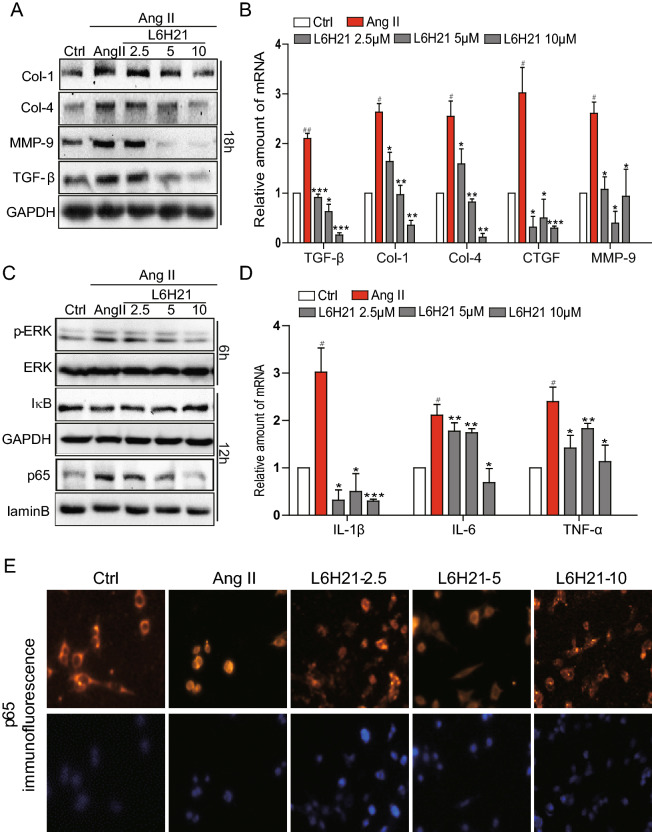
L6H21 pretreatment prevents Ang II-induced fibrosis and signal activation in renal tubular epithelial cells. The effects of the MD2 inhibitor L6H21 on Ang II-stimulated inflammatory responses in the renal tubular epithelial cell line, NRK-52E, were determined. NRK-52E were pretreated with the vehicle control DMSO (Ctrl) or L6H21 (2.5, 5.0, 10 μM) for 1 h, and stimulated with Ang II (1 μM) for different periods. (**A**) Representative Western blot analysis for protein markers of fibrosis, Col-1 (collagen 1), Col-4 (collagen 4), MMP-9 (metalloproteinase 9), and TGF-β (transforming growth factor β), GAPDH as loading control; n = 3 independent determinations. (**B**) The mRNA levels of TGF-β, Col-1 (collagen 1), Col-4, CTGF, and MMP-9 were detected by real-time qPCR; values normalized to house-keeping gene β-actin. (**C**) Representative Western blot analysis for p-ERK (phosphorylated ERK) and IκB from total cell lysate, GAPDH as loading addingol, ERK = total ERK; NF-κB p65 subunit detected from nuclear cell fraction, lamin B as loading control; n = 3 independent determinations. (**D**) The mRNA levels of IL-1β, IL-6 and TNF-α were detected by real-time qPCR; values normalized to house-keeping gene β-actin. (**E**) Representative immunofluorescent distribution of NF-κB p65 subunit (Texas-red streptavidin), top row; same cells counterstained with nuclear stain DAPI, bottom row, 200× magnification; n = 3 independent determinations. Data in B and D are reported as mean ± S.E.M. of n = 3, *P < 0.05, **P < 0.01, ***P < 0.001 versus Ang II-treated group; ^#^P < 0.05 and ^##^P < 0.01 versus Ctrl. For panels (**A**,**C**) the gels were run under the same experimental conditions. Shown are cropped gels/blots (The gels/blots with indicated cropping lines are shown in Supplementary Figure S6).

