# Supplementary material for: Angiotensin II induces kidney inflammatory injury and fibrosis through binding to myeloid differentiation protein-2 (MD2)
Source: Sci Rep. 2017 Mar 21;7:44911. doi: 10.1038/srep44911 (PMC5359637; doi:10.1038/srep44911)
Supplement: Supplementary Information [file srep44911-s1.doc]

**Supplementary information**

## Angiotensin II induces kidney inflammatory injury and fibrosis through binding to myeloid differentiation protein-2 (MD2)

Zheng Xu^1,#^, Weixin Li^1,#^, Jibo Han^1,2^, Chunpeng Zou^3^, Weijian Huang^2^, Weihui Yu^2^, Xiaoou Shan^3^, Hazel Lum^1^, Xiaokun Li^1^,*, Guang Liang^1^,*

*^1^ Chemical Biology Research Center, School of Pharmaceutical Sciences,* *Wenzhou Medical University, Wenzhou, Zhejiang, China;*

*^2^* *The First Affiliated Hospital, Wenzhou Medical University, Wenzhou, Zhejiang, China;*

*^3^* *The Second Affiliated Hospital, Wenzhou Medical University, Wenzhou, Zhejiang, China*

**Supplementary Table S1.** MD2 deficiency did not change Ang II-increased systolic blood pressure (SBP) in mice. The animal experiment was carried out as described in Methods and Materials and the SBP was detected at day 0, week 4^th^ and week 8^th^ after Ang II treatment by non-invasive tail-cuff Pressure Analysis System (Softron BP-98A, Tokyo, Japan). (n=8; ^#^ P<0.05, vs Ctrl group; ns = no significant, vs Ang II group).

|  | **Ctrl** | **Ang II** | **MD-2^-/-^** | **MD-2^-/-^ +Ang II** |
| --- | --- | --- | --- | --- |
| **Week 0** | 92.96 ± 9.39 | 88.52 ± 10.25 | 93.54 ± 4.562 | 94.50 ± 8.771 |
| **Week 4** | 95.19 ± 8.873 | 124.1 ± 18.69^#^ | 94.53 ± 3.429 | 129.2 ± 22.53^#, ns^ |
| **Week 8** | 89.26 ± 7.936 | 131.3 ± 10.32^#^ | 90.25 ± 5.454 | 131.7 ± 23.49^#, ns^ |

**Supplementary Table S2.** Primer sequences for real-time quantitative PCR.

| **Gene** | **Species** | **Forward primer** | **Reverse primer** |
| --- | --- | --- | --- |
| TNF-α | Rat | TACTCCCAGGTTCTCTTCAAGG | GGAGGCTGACTTTCTCCTGGTA |
| IL-6 | Rat | GAGTTGTGCAATGGCAATTC | ACTCCAGAAGACCAGAGCAG |
| IL-1β | Rat | GGGCCTCAAGGGGAAGAATC | ATGTCCCGACCATTGCTGTT |
| Col-1 | Rat | GACATCCCTGAAGTCAGCTGC | TCCCTTGGGTCCCTCGAC |
| Col-4 | Rat | TGGCCTTGGAGGAAACTTTG | CTTGGAAACCTTGTGGACCAG |
| TGF-β | Rat | AGGAGGAATTTGGCCAGGTG | GCTCACGAGGAGGCTAATCC |
| MMP-9 | Rat | AGCTGGCAGAGGCATACTTGT | GTGCTCCGCGACACCAAACTG |
| CTGF | Rat | GCCTGTTCCAAGACCTGT | GGATGCACTTTTTGCCCTTCTTA |
| β-actin | Rat | AAGTCCCTCACCCTCCCAAAAG | AAGCAATGCTGTCACCTTCCC |

**Supplementary Figure S1**. Densitometric quantification for Figure 1D; values normalized to GAPDH. Data are reported as mean ± s.e.m. from 4 mouse tissues per group and analyzed by Student’s t-test; # p<0.05, v.s. Ctrl group; * p < 0.05 and ** p < 0.01, v.s. Ang II group.

**Supplementary Figure S2**. Knockdown of MD2 (**A**) and TLR4 (**B**) in NRK-52E cells by siRNA transfection. Figures showing protein levels of MD2 and TLR4 in untransfected cells (Ctrl) and in cells transfected with either control negative siRNA (siCtrl) or siRNA targeting MD2 (siMD2) and TLR4 (siTLR4), respectively (n=4 experiments).

**Supplementary Figure S3**. The effects of MD2 inhibitor L6H21 and AT1 blocker Irbsatan (IRB) on Ang II-induced MD2/TLR4 complex and inflammation in NRK-52E cells. A. NRK-52E cells were pre-treated with L6H21 (10 μM), IRB (10 μM) or vehicle for 1h and then stimulated with Ang II (1 μM) for 30min, and cell lysates were co-immunoprecipitated (IP) with anti-MD2 antibody, and western blot analysis (IB) made to detect TLR4; n=3 independent determinations. B and C. NRK-52E cells were pre-treated with L6H21 (10 μM), IRB (10 μM) or vehicle for 1 h and then stimulated with Ang II (1 μM) for 24 h. The protein levels of IL-6 (B) and TNF-α (C) and in the culture medium were measured by ELISA and normalized to the total amount of protein respectively. (Data from 3 independent experiments; ns = not significant, v.s. Ang II group; ** P<0.001, vs Ang II group; # P<0.05, ### P<0.001, vs Ctrl group).

**Supplementary Figure S4**. The effects of MD2 inhibitor L6H21on Ang II-induced MD2/TLR4 complex and inflammation in different cells. A-C. SV40 cells were pre-treated with L6H21 (10 μM) or vehicle for 1h and then stimulated with Ang II (1 μM) for 30min, and cell lysates were co-immunoprecipitated (IP) with anti-MD2 or anti-TLR4 antibodies, and western blot analysis (IB) made to detect TLR4 or MyD88, respectively; n=3 independent determinations. B and C. SV40 cells were pre-treated with L6H21 (10 μM), IRB (10 μM) or vehicle for 1 h and then stimulated with Ang II (1 μM) for 24 h. The protein levels of IL-6 (B) and TNF-α (C) and in the culture medium were measured by ELISA and normalized to the total amount of protein respectively. D-F. The same experiments as A-C were performed in HMEC-1 cells. G-H. The same experiments as B-C were performed in mouse macrophages. (Data from 3 independent experiments; *P<0.05 and ** P<0.001, vs Ang II group; # P<0.05, ### P<0.001, vs Ctrl group).

**Supplementary Figure S5. Ang II interacts MD2. A)** Cell-free assay was made to evaluate Ang II binding to purified human recombinant MD2 (rhMD2). Recombinant human MD2 (rhMD2) protein and recombinant human TLR4 (rhTLR4) protein was purchased from R&D System (Minneapolis, MN, USA). Ang II labelled with biotin was purchased from GL Biochem Ltd. (Shanghai, China). The rhMD2 (4 mg/ml) was incubated with anti-rhMD2 antibody pre-coated on an ELISA plate, biotin-labeled Ang II (400, 200, 100, 50, 25 μM) was subsequently added for incubation of 1 h, and evaluation of direct binding with rhMD2 by measures of absorbance (OD) at 450 nm; biotin-labeled LPS (bio-LPS) as positive control; OD values reported as mean±S.E.M. n=3, each performed in duplicate; ***P＜0.001 versus control (Ctrl) buffer alone. **B)** Cell-free ELISA for evaluation of Ang II binding to purified rhMD2, using the same method as described for C; for this, 100 μM of bio-Ang II was incubated with or without L6H21 (0.1, 1, 10 μM). OD values are reported as mean±S.E.M., n=3 (with each performed in duplicates); ^###^P＜0.001 versus Ctrl, *P＜0.05, **P＜0.01, ***P＜0.001 versus Bio-Ang II group. **C)** The binding affinity of Ang II to rhMD2 was determined using a ProteOn XPR36 Protein Interaction Assay system (The methodology is described in our previous paper “*Basic Research in Cardiology*, 2017, 112: 9”); shown is a representative graph of the effects of 48.8 μM – 50 mM Ang II; the binding kinetics and KD indicated on upper left corner. **D)** The binding affinity assay of Ang II to rhTLR4 determined using a ProteOn XPR36 system showed no interactions between Ang II and TLR4 protein using the same method.

**Supplementary Figure S6**. Gels/blots with the cropping lines.
